# Prompt Thrombo-Inflammatory Response to Ischemia-Reperfusion Injury and Kidney Transplant Outcomes

**DOI:** 10.1016/j.ekir.2023.09.025

**Published:** 2023-09-24

**Authors:** Gabriel Strandberg, Carl M. Öberg, Anna M. Blom, Oleg Slivca, David Berglund, Mårten Segelmark, Bo Nilsson, Ali-Reza Biglarnia

**Affiliations:** 1 Department of Surgery, Department of Clinical Sciences Malmö, Skåne University Hospital, Lund University, Malmö, Sweden; 2 Department of Nephrology, Department of Clinical Sciences Lund, Skåne University Hospital, Lund University, Lund, Sweden; 3 Department of Translational Medicine, Lund University, Malmö, Sweden; 4 Department of Immunology, Genetics, and Pathology (IGP), Rudbeck Laboratory C5:3, Uppsala University, Uppsala, Sweden

**Keywords:** delayed graft function, intravascular innate immune system, ischemia-reperfusion injury, kidney transplantation, organ preservation, thromboinflammation

## Abstract

**Introduction:**

In kidney transplantation (KT), the role of the intravascular innate immune system (IIIS) in response to ischemia-reperfusion injury (IRI) is not well-understood. Here, we studied parallel changes in the generation of key activation products of the proteolytic cascade systems of the IIIS following living donor (LD) and deceased donor (DD) transplantation and evaluated potential associations with clinical outcomes.

**Methods:**

In a cohort study, 63 patients undergoing LD (*n* = 26) and DD (*n* = 37) transplantation were prospectively included. Fifteen DD kidneys were preserved with hypothermic machine perfusion (HMP), and the remaining were cold stored. Activation products of the kallikrein-kinin, coagulation, and complement systems were measured in blood samples obtained systemically at baseline and locally from the transplant renal vein at 1, 10, and 30 minutes after reperfusion.

**Results:**

DD kidneys exhibited a prompt and interlinked activation of all 3 cascade systems of IIIS postreperfusion, indicating a robust and local thrombo-inflammatory response to IRI. In this initial response, the complement activation product sC5b-9 exhibited a robust correlation with other IIIS activation markers and displayed a strong association with short-term and mid-term (24-month) graft dysfunction. In contrast, LD kidneys did not exhibit this thrombo-inflammatory response. The use of HMP was associated with reduced thromboinflammation and preserved mid-term kidney function.

**Conclusion:**

Kidneys from DD are vulnerable to a prompt thrombo-inflammatory response to IRI, which adversely affects both short-term and long-term allograft function. Strategies aimed at minimizing graft immunogenicity prior to reperfusion are crucial to mitigate the intricate inflammatory response to IRI.

## Graphical abstract

**Figure:**
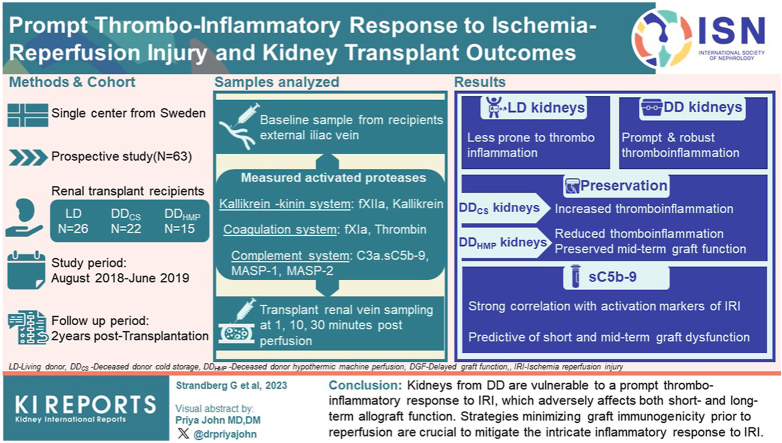

The IIIS is a composite entity that consists of the proteolytic cascades of the complement, coagulation, kallikrein-kinin (contact system), and fibrinolytic systems, along with blood effector cells such as granulocytes, monocytes, and platelets in this crosstalk.^1^ In contrast to the adaptive immune system, the IIIS can distinguish self from nonself at the species level but not at the individual level. This preset feature is not only relevant for defense against invasive pathogens but also enables the IIIS to react to both autologous and allogeneic cells, which is essential for its role in medical conditions and interventions.^2^

Solid organ transplantation comprises a series of coordinated steps from donor management and procurement to organ preservation and engraftment. These sequential events require a temporary impairment and cessation of blood supply to organs, thus causing a deprivation of oxygen delivery, that is, ischemia, which is a major stress to the cells. In addition to the creation of an acidic environment and upregulation and downregulation of several genes, which results in a proinflammatory state, ischemic stress can alter the phenotype of cells by changing their membrane composition and protein expression.^3^, ^4^, ^5^, ^6^ For example, the disintegration of the glycocalyx with subsequent loss of antithrombotic and anti-inflammatory regulators on the endothelial cell surface is a critical event induced by ischemia that exposes the “naked” endothelial cells to attacks from the IIIS and further damage.^4^

The restoration of the blood supply, that is, reperfusion to organ grafts, is a necessary step. Reperfusion is the starting point for healing from hypoxic stress, thereby ensuring organ graft viability and function. However, depending on the extent of hypoxic injury, the direct interaction between altered cell lining and IIIS following reperfusion can initiate a response that misfires, causing an inadvertent response that causes strong activation of proteolytic cascades and, by extension, IRI. Similarly, when the IIIS reacts to altered autologous cells with different phenotypes than the native undamaged self, IRI can also occur in other medical conditions, such as stroke, myocardial infarction, or sepsis.^4^

In clinical KT, complement activation is traditionally considered the major effector response during IRI-induced inflammation. Evidence, most of which comes from animal studies, points to early complement activation following reperfusion that has been linked to early and late graft dysfunction.^7^, ^8^, ^9^, ^10^, ^11^ However, in clinical trials, the use of complement therapeutics to attenuate IRI-induced graft injury has not yielded strong conclusions.^12^, ^13^, ^14^

Here, we assumed that in kidney allografts exposed to ischemia, the direct attack by IIIS could trigger the coactivation of multiple proteolytic cascades as a common system, resulting in a multifaceted inflammatory response to IRI. To assess this, we conducted a prospective cohort study that investigated simultaneous changes in key activation products of the kallikrein-kinin, coagulation, and complement systems in early samples obtained from the transplant renal vein during consecutive minutes after reperfusion. All patients were followed-up with for 24 months to evaluate short-term and mid-term consequences of the initial inflammatory response to IRI.

## Methods

### Study Population

The study enrolled all 63 uremic patients (44 males and 19 females) who underwent sequential KT at the Department of Transplantation, Skåne University Hospital, Sweden, between August 2018 and June 2019. Twenty-six patients were recipients of LD kidneys, and 37 patients were recipients of DD kidneys, of which 22 kidneys were preserved with static cold storage (DD_CS_) and 15 were preserved with HMP (DD_HMP_) (LifePort Kidney Transporter, Itasca, Illinois). At our center, the donation-after-circulatory-death modality was implemented after the study period; therefore, the DD population exclusively consisted of donation-after-brain-death donors. Per standard of care, 61 of 63 patients received induction treatment with basiliximab and methylprednisolone. Two patients (DD_CS_) received induction with thymoglobulin due to human leukocyte antigen immunization. One patient (DD_HMP_) received rituximab in combination with basiliximab on day 0, due to ABO-incompatibility. All patients received the same maintenance immunosuppressive regimen combining tacrolimus, mycophenolate mofetil, and prednisolone, following the widely implemented SYMPHONY protocol.^15^

Data on donor/recipient characteristics and outcome parameters (serum creatinine, estimated glomerular filtration rate [eGFR], delayed graft function (DGF), graft failure, *de novo* donor-specific antibodies, biopsy-proven acute rejections, and patient death) were retrieved from patient records, the ScandiaTransplant database and the local transplant registry for a total follow-up period of 24 months post-transplantation. Cold ischemic time (CIT) was defined as the duration between *in situ* (in DD) or *ex situ* (in LD) cold perfusion and established graft reperfusion in recipients. The eGFR (ml/min per 1.73 m^2^) was calculated using the Lund-Malmö revised formula.^16^ DGF was defined as the need for dialysis within the first week post-transplantation. The study was approved by the Regional and National Ethical Committee (DNR 2017-79), and written informed consent was obtained from all patients.

### Sample Collection

Six milliliter whole-blood aliquots were drawn from the kidney vein into EDTA vacutainer tubes (K2E BD Vacutainer, Becton, Dickinson and Company) during the transplant procedure. An initial baseline sample was obtained preimplantation by venipuncture of the recipient’s external iliac vein at the transplant site followed by 3 sequential samples drawn from the transplant vein at 1, 10, and 30 minutes postreperfusion. To minimize the risk of unintentional activation of the proteolytic cascades, samples were drawn directly into EDTA-anticoagulated tubes by venipuncture with a butterfly needle system (21G BD Vacutainer UltraTouch, Becton, Dickinson and Company) and immediately cooled in ice-sludge. The transplant vein was punctured in its proximal portion with the needle tip directed toward the hilum to minimize contamination with systemic venous blood. Needles were extracted and disposed of after each venipuncture. Samples were centrifuged at 1900*g* for 10 minutes at 4 °C to separate the plasma portion. The plasma was extracted, aliquoted, and stored at −80 °C.

### Sample Analyses

All samples were assigned numeric codes to ensure sample analysis in a blinded fashion. In addition, staff analyzing samples were kept unaware of the study design and purpose. The proteolytic cascade systems of the blood consist of proteases whose zymogens, for example, prothrombin and prekallikrein, are activated by an upstream protease. Many of their inhibitors are serpins, for example, C1-inhibitor (C1INH) and antithrombin (AT), which form complexes with the protease. This allows analyses of the activation of individual zymogens if the zymogen-serpin complex is quantified in blood plasma.^17^ The following activated proteases were measured in complexes with both serpins AT and C1INH: (i) kallikrein-kinin system: factor XIIa (FXIIa-AT and FXIIa-C1INH), kallikrein (KK-AT and KK-C1INH); (ii) coagulation system: factor XIa (FXIa-AT and FXIa-C1INH), thrombin (thrombin-AT and thrombin-C1INH); and (iii) complement system: mannan-binding lectin serine protease 1 (MASP1-AT and MASP1-C1INH), mannan-binding lectin serine protease 2 (MASP2-AT and MASP2-C1INH). In-house analyses were performed according to previously described methods^17^^,^^18^ using Luminex xMAP Technology (Merck Millipore) on a clinical diagnostics instrument (MAGPIX, Luminex Corporation). Soluble terminal complement activation fragment (sC5b-9) and complement protein C3a (C3a) were assessed using in-house ELISAs.^19^

### Statistical Analysis

Continuous variables are expressed as medians (interquartile ranges), and categorical variables as frequencies (percentages). Kruskal‒Wallis tests with Bonferroni correction or Mann‒Whitney U and chi-square or Fisher exact tests were used to assess differences in characteristics, outcome parameters, and eGFR slopes. Multiple comparison analysis was performed using analysis of variance on aligned rank-transformed data. Simple linear regression was used to assess slopes for eGFR from 1 to 24 months post-transplantation. Associations of outcome parameters or CIT with IIIS activation markers were performed using receiver operating characteristic curve analyses. Spearman’s correlation analysis was employed to estimate correlations between IIIS markers within and across cascade systems. A *P*-value of less than 0.05 was considered significant. Statistical analyses were performed using GraphPad Prism 9.2.0, GraphPad Software, San Diego California USA; IBM SPSS Statistics 28, IBM Corp., Armonk, NY; and R version 4.1.1, for Mac, R Foundation for Statistical Computing, Vienna, Austria.

## Results

There were no differences in sex (referred to different biological characteristics of female and male), recipient/donor body mass index, preformed donor-specific antibodies, cause of kidney failure, and induction treatment between the DD-KT and LD-KT groups. In the DD-KT population, there was no difference in the kidney donor risk index between DD_CS_ and DD_HMP_. Recipient/donor age and CIT were higher in DD-KT than in LD-KT. The overall time of pretransplant dialysis was shorter in LD-KT than in DD-KT.

The overall median CIT in DD-KT was restricted to 692 minutes (interquartile range [IQR] 547–902). Between the DD_CS_-KT and DD_HMP_-KT subgroups, there were no differences in median CIT (684 [IQR 547–858] vs. 781 [IQR 504–1029] minutes, *P* = 0.53) and kidney donor risk index (1.56 [IQR 1.28–1.97] vs. 1.50 [IQR 1.28–1.97], *P =* 0.92) (Table 1). There was one case of patient death (with a functioning graft) in DD_CS_-KT on day 64 post-transplantation due to tissue-invasive cytomegalovirus disease. There were 2 graft failures post-DD_CS_-KT (due to acute antibody-mediated rejection) and DD_HMP_-KT (recurrence of thrombotic microangiopathy) on days 151 and 396, respectively. In LD-KT, 1 graft failure occurred due to recurrence of focal segmental glomerulosclerosis on day 469 post-transplantation. At the last follow-up, no differences were observed in overall transplant function, the incidence of biopsy-proven acute rejections, and both graft and patient survival (Table 2). One LD-KT patient was lost to follow-up on day 69 due to emigration. There were no surgical complications related to intraoperative venipuncture of the kidney allograft.

**Table 1:** Baseline characteristics of the study population separated by KT subgroups LD, cold stored DD (DD_CS_), and hypothermic machine perfused DD (DD_HMP_)

Baseline characteristics-median (1st–3rd quartile) or *n* (%)	Transplant modality (63)			*P* value
	LD (26)	DD (37)		
		DD_CS_ (22)	DD_HMP_ (15)	
Donor age	51.0 (46.0–59.0)	56.5 (49.8–67.8)	62.0 (46.0–68.0)	0.036^a^
Donor BMI	26.4 (23.5–29.3)	24.8 (22.2–29.5)	23.6 (22.2–29.7)	ns
Donor anoxic brain injury (prior cardiac arrest)	-	6 (27.2)	7 (46.7)	ns
Kidney donor risk index	-	1.56 (1.28–1.97)	1.50 (1.28-1.97)	ns
Cold ischemic time in minutes	116.5 (95.0–154.8)	684.0 (546.8–858.0)	781.0 (504.0–1029.0)	<0.001^a^^,^^b^^,^^c^
Recipient sex–male	18 (69.2)	14 (63.6)	12 (80.0)	ns
Recipient age	45.5 (35.8–54.3)	53.5 (46.0–60.5)	60.0 (45.0–66.0)	0.011^a^, 0.037^c^
Recipient BMI	25.9 (22.5–28.0)	26.0 (23.8–28.5)	24.9 (23.1–26.9)	ns
Preformed DSA	4 (15.4)	4 (18.2)	2 (13.3)	ns
Induction treatment				
Basiliximab	26 (100.0)	20 (90.9)	14 (93,3)	ns
Basiliximab + Rituximab	0 (0.0)	0 (0.0)	1 (6.7)	ns
Thymoglobulin	0 (0.0)	2 (9.1)	0 (0.0)	ns
Pretransplant dialysis modality				
Predialytic	11 (42.3)	3 (13.6)	1 (6.7)	0.006^a^, 0.03^c^
Hemodialysis	9 (34.6)	15 (68.2)	9 (60.0)	0.023^a^, 0.04^b^
Peritoneal dialysis	6 (23.1)	4 (18.2)	5 (33.3)	ns
Days of pretransplant dialysis	392 (182–730)	1529 (683–1924)	785 (539–1354)	<0.001^a^, 0.001^b^
Cause of kidney failure				
Glomerulonephritis	14 (53.8)	5 (22.7)	6 (40.0)	0.027^b^
Diabetic nephropathy	3 (11.5)	5 (22.7)	1 (6.7)	ns
Polycystic kidney disease	3 (11.5)	3 (13.6)	1 (6.7)	ns
Hypertensive nephrosclerosis	3 (11.5)	2 (9.1)	3 (20.0)	ns
Alport syndrome	1 (3.8)	3 (13.6)	0 (0.0)	ns
Unknown	0 (0.0)	1 (4.5)	3 (20.0)	0.043^c^
Other	2 (7.7)	3 (13.6)	1 (6.7)	ns

BMI, body mass index; DD, deceased donor; DD_CS_, deceased donor kidney preserved with static cold storage; DD_HMP_, deceased donor kidney preserved with hypothermic machine perfusion; DSA, donor-specific antibodies; LD, living donor.

Data are presented as frequencies (column percentages) and medians (1st–3rd quartile). P-values are expressed for specified comparisons:

ns = not significant.

a LD vs. DD.

b LD vs. DD_CS_.

c LD vs. DD_HMP_.

**Table 2:** Outcome parameters of the study population separated by KT subgroups LD, cold stored DD (DD_CS_), and hypothermic machine perfused DD (DD_HMP_)

Outcome at 24 months median (1st–3rd quartile) or *n* (percent)	Transplant modality (63)			*P*-value
	LD (26)	DD (37)		
		DD_CS_ (22)	DD_HMP_ (15)	
s-Creatinine μmol/l	103.0 (93.8–138.5)	117.5 (96.3–149.0)	130.0 (103.0–155.0)	ns
eGFR ml/min per 1.73 m^2^	62.0 (49.0–69.8)	56.5 (34.3–68.0)	45.5 (34.8–64.8)	ns
Graft failure	1 (3.8%)	2 (9.1%)	1 (6.7%)	ns
Delayed graft function	0 (0.0%)	4 (18.2%)	2 (13.3%)	0.038^a^^,^^b^
Patient death	0 (0.0%)	1 (4.5%)	0 (0.0%)	ns
Biopsy-proven acute rejection	7 (26.9%)	6 (27.3%)	5 (33.3%)	ns
De novo DSA	3 (11.5%)	3 (13.6%)	0 (0.0%)	ns

DD, deceased donor; DSA, donor-specific antibodies; DD_CS_, deceased donor kidney preserved with static cold storage; DD_HMP_, deceased donor kidney preserved with hypothermic machine perfusion; LD, living donor.

Data are presented as frequencies (column percentages) and medians (1st–3rd quartile). *P*- values are expressed for specified comparisons:

ns = not significant.

a LD vs. DD.

b LD vs. DD_CS_.

### Generation of Key Activation Markers of IIIS During IRI

**Figure 1:**
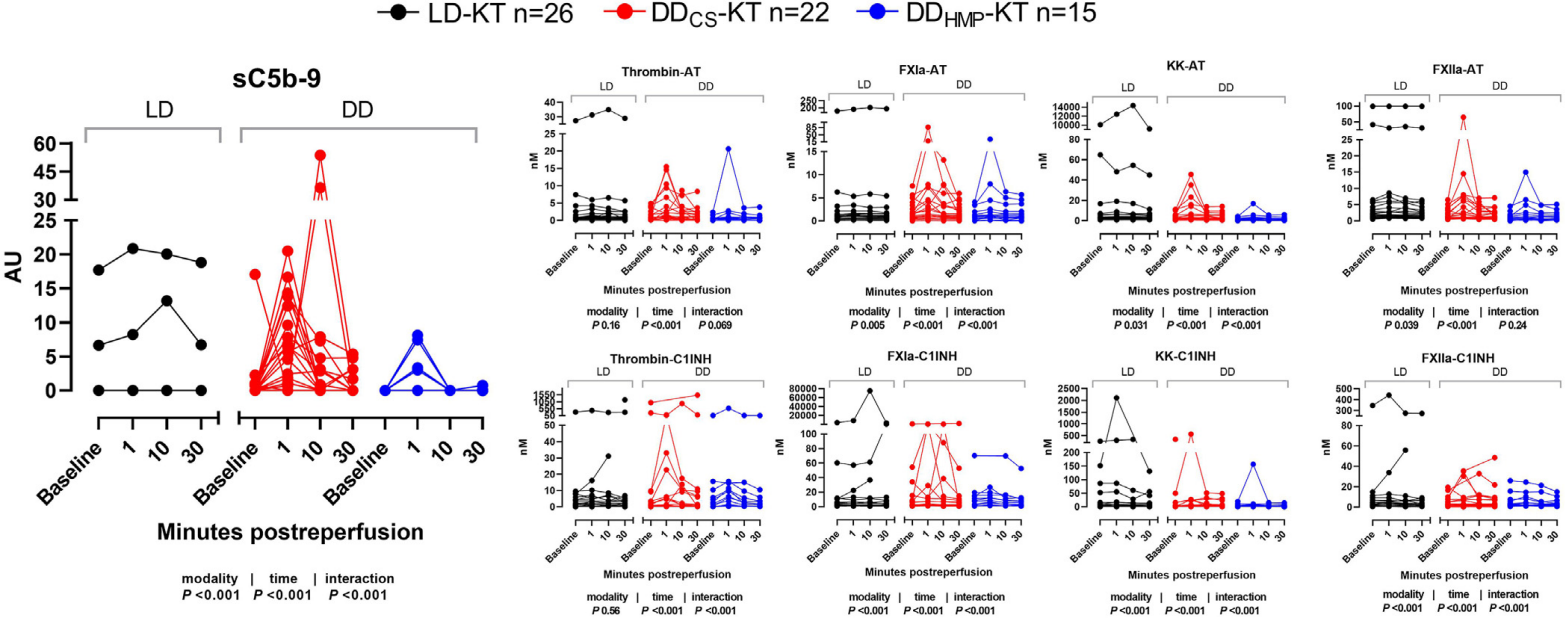
Levels of activation markers of the IIIS stratified by modality and time for the study population (*n* = 63). Connected scatter plots are color-coded for the specified modalities; black = LD-KT, red = DD_CS_-KT and blue = DD_HMP_-KT. *P*-values for differences from analysis of variance on aligned rank-transformed data by modality (LD-KT [*n* = 26], DD_CS_-KT [*n* = 22] and DD_HMP_-KT [*n* = 15]), time (baseline, 1, 10, and 30 minutes postreperfusion), and the interaction (between time and modality) are presented below each graph (ns = not significant). DD_CS_, deceased donor kidney preserved with static cold storage; DD_HMP_, deceased donor kidney preserved with hypothermic machine perfusion; LD, living donor.

The relationships between IIIS activation markers and the 2 independent variables, time (baseline and 1, 10, and 30 minutes after reperfusion) and modality (LD-KT, DD_CS_-KT, and DD_HMP_-KT), and their interaction were analyzed by an analysis of variance on aligned rank-transformed data. To maintain statistical power, the various parameters were not normalized to baseline values. This decision was made considering that repeated measures analysis of variance already accounts for baseline values. Factors representing activation of complement (sC5b-9), coagulation (FXIa-AT and FXIa-C1INH), and kallikrein-kinin systems (KK-AT, KK-C1INH, and FXIIa-C1INH) were dependent on time and modality. Furthermore, the combined effect of time and modality on the activation of these markers was indicated by significant interactions. The downstream activation of the coagulation system indicated by thrombin-AT and thrombin-C1INH complexes showed a relationship with time and an interaction effect of time and modality for thrombin-C1INH. FXIIa-AT levels were associated with time and modality, but no interaction effect was observed. Figure 1 shows activation markers of IIIS with a significant interaction in either or both of their respective protease-serpin complexes. The complete dataset is presented in Supplementary Table S1.

### Correlations of Markers Within and Between the Proteolytic Cascades of IIIS

**Figure 2:**
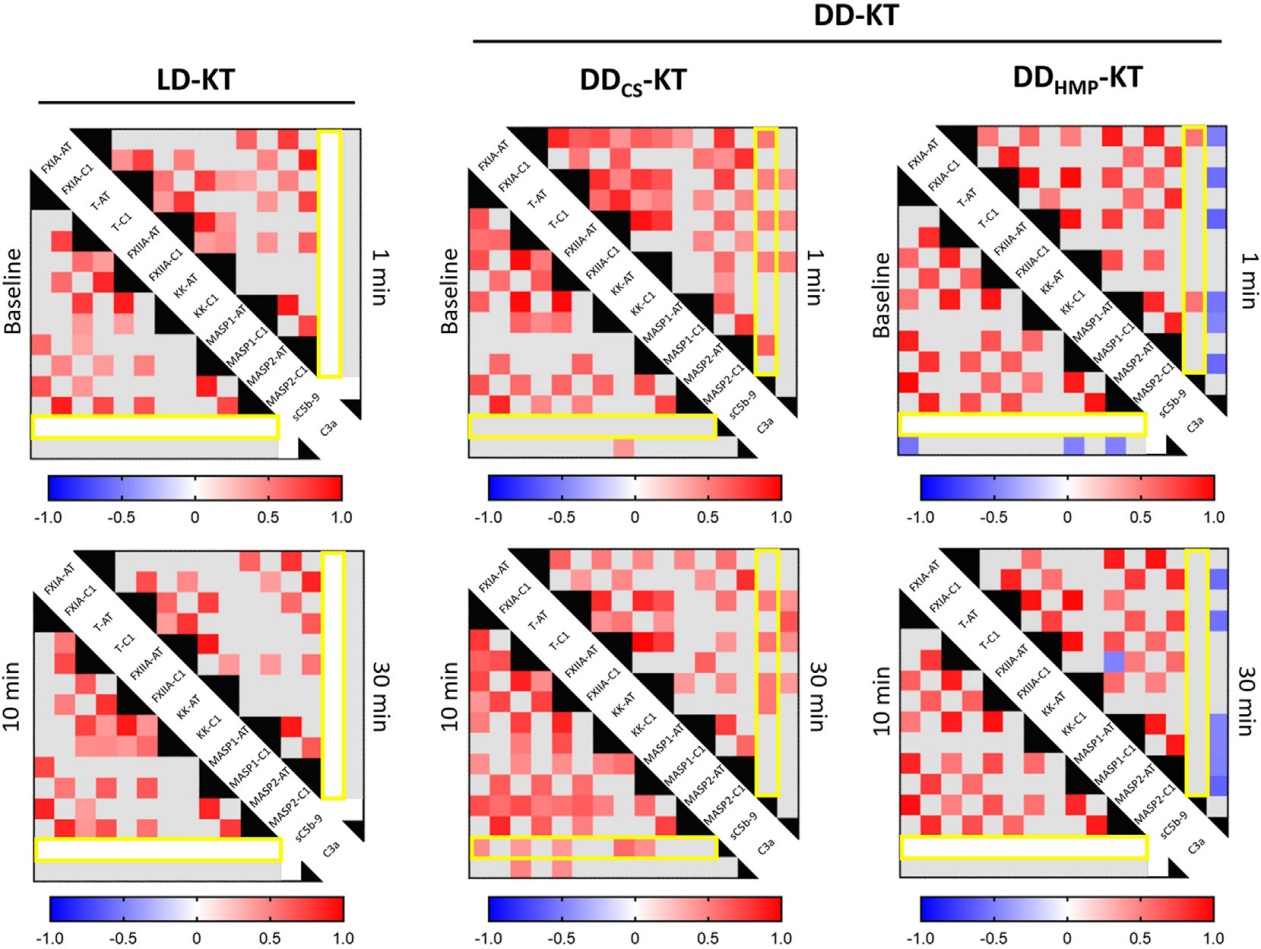
Spearman correlation matrices by modality (DD separated by preservation method) from baseline to 30 minutes postreperfusion. The red gradient denotes significant positive *ρ* coefficients, and blue denotes significant negative *ρ* coefficients. Gray squares denote nonsignificant correlations. White squares indicate incomputable correlations due to non or insufficient generation of sC5b-9. A yellow outline highlights sC5b-9. DD_CS_, deceased donor kidney preserved with static cold storage; DD_HMP_, deceased donor kidney preserved with hypothermic machine perfusion; LD, living donor.

Spearman’s correlation analysis was performed to assess the relationship between IIIS activation markers within and across the proteolytic cascades. Apart from strong overall correlations within each cascade system, the pattern of cross-cascade correlations differed between modalities (LD-KT, DD_CS_-KT, and DD_HMP_-KT) and/or time (Figure 2). This difference was most evident in the generation of the complement activation product sC5b-9. In LD kidneys, sC5b-9 generation was absent in 24 out of 26 cases (92.3%) throughout the sampling time. Consequently, calculations on sC5b-9 correlations were not performed in LD-KT. Furthermore, correlations within and between the proteolytic cascades remained largely unchanged in LD-KT postreperfusion. Notably, sC5b-9 generation was detected at baseline and throughout the sampling period in only 2 out of 26 LD-KT cases. Therefore, the confounding effect of these 2 individuals was deemed to be minimal.

Although absent at baseline, the generation of sC5b-9 in DD_CS_ kidneys was promptly accompanied by activation of MASP-2 (*ρ* 0.65, *P* = 0.001), FXIa (*ρ* 0.57, *P* = 0.005), thrombin (*ρ* 0.53, *P* = 0.012), FXIIa (*ρ* 0.54, *P* = 0.01), and kallikrein (*ρ* 0.54, *P* = 0.009) at 1-minute postreperfusion, which indicated prompt coactivation of the complement, coagulation, and kallikrein-kinin systems and, by extension, a thrombo-inflammatory response that was retained throughout the sampling period. In DD_HMP_ kidneys, sC5b-9 generation was correlated with the activation of FXIa (*ρ* 0.58, *P* = 0.024) and MASP-1 (*ρ* 0.58, *P* = 0.023) at 1-minute postreperfusion; however, these correlations were not retained throughout the sampling time. This indicated a marked attenuation of the thrombo-inflammatory response by HMP. A complete correlation dataset is provided in Supplementary Table S2A–4D.

### Associations Between sC5b-9 Generation, CIT, DGF, and Mid-Term Graft Function

Soluble C5b-9 was considered as a response marker for IIIS activation based on the following 2 observations: (i) concurrent generation of sC5b-9 and other IIIS activation markers in response to IRI and (ii) attenuation of IIIS activation in DD_HMP_ kidneys corresponded to a reduction in sC5b-9 generation. This consideration was also relevant to prevent spurious correlations that would have occurred when testing for various markers of IIIS individually.

To evaluate the impact of IIIS activation on DGF and mid-term kidney function, we tested for associations when accounting for sC5b-9 generation at different time points and for the “area under the curve” (AUC) value of sC5b-9 from baseline to 30 minutes (AUC_b-30_) for all patients. The eGFR slope was calculated to assess the mid-term (24 months) transplant function for all patients.

**Figure 3:**
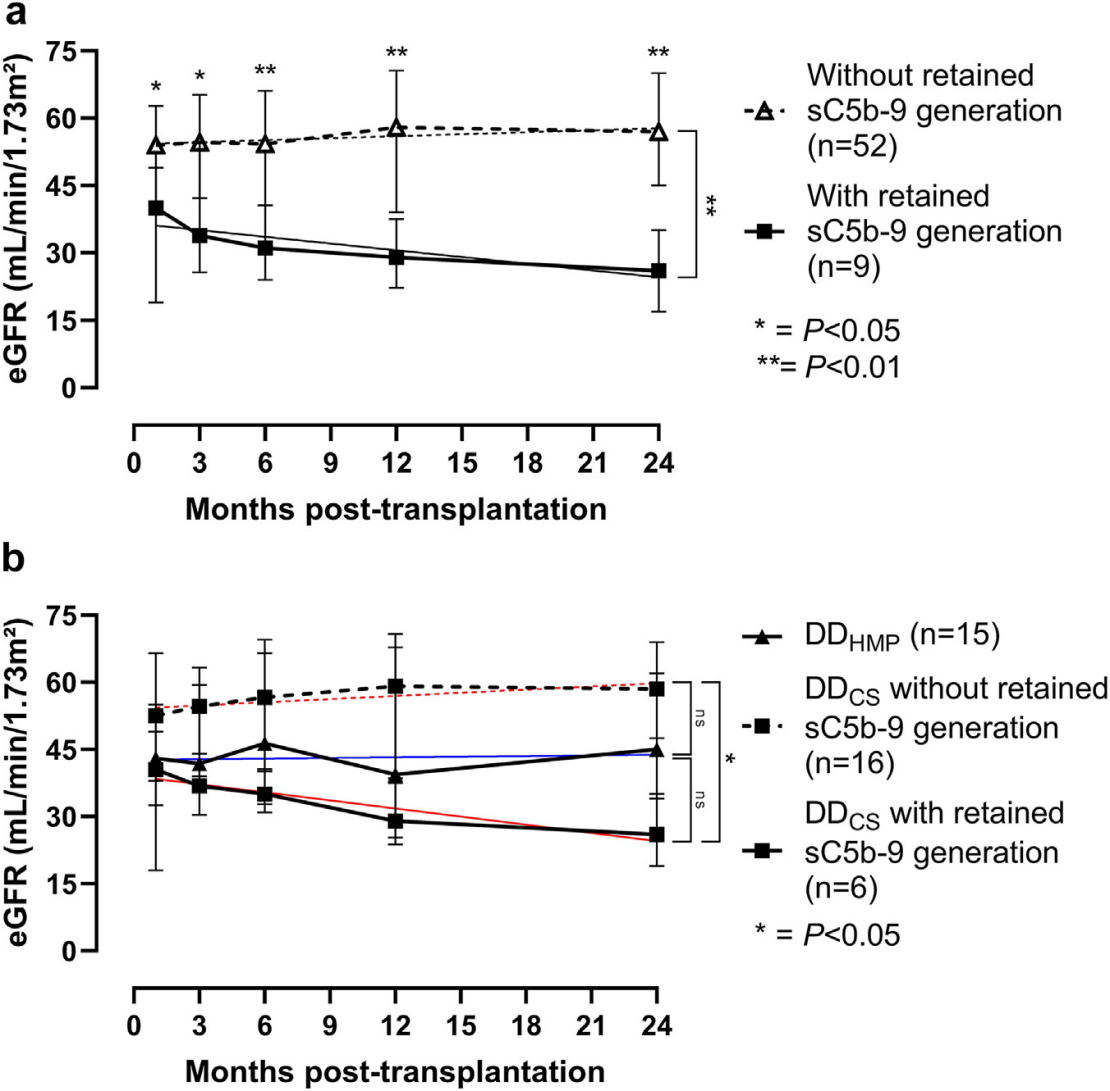
Progression of median eGFR (ml/min per 1.73 m^2^) with interquartile range from 1 to 24 months post-transplantation stratified (a) for retained sC5b-9 generation (defined as sC5b-9 generation at 30 minutes following reperfusion) within the study cohort and (b) for modalities DD_HMP_ and DD_CS_ with and without retained sC5b-9 generation. The eGFR slopes, calculated between 1 and 24 months, are represented by filled and dashed lines in the figures. The lines are color-coded in red for DD_CS_ and blue for DD_HMP_. Two LD-KT patients with missing data on 30-minute sC5b-9 are excluded from the analyses. In addition, 2 patients (DD_CS_-KT and LD-KT) are excluded from the slope analyses due to patient death on day 64 and loss to follow-up on day 69, respectively. DD_CS_, deceased donor kidney preserved with static cold storage; DD_HMP_, deceased donor kidney preserved with hypothermic machine perfusion; eGFR, estimated glomerular filtration rate.

The sC5b-9 AUC_b−30_ showed a significant association with DGF (receiver operating characteristic AUC 0.81 [95% CI 0.61–1.00, *P* = 0.012]). Considering the impact of HMP on retained IIIS activation in Spearman’s correlation analysis, a stratification for retained IIIS response (defined as sC5b-9 generation at 30 minutes postreperfusion) showed that the subgroup of patients with retained sC5b-9 activity (LD-KT 2/26, DD_CS_-KT 6/22 and DD_HMP_-KT 1/15) had lower eGFR levels throughout the 24-month follow-up period with median eGFR (IQR) differences between those with retained sC5b-9 and those without retained sC5b-9 generation at 1 month: 40.0 (20.0–49.0) versus 54.0 (40.0–62.5) *P* = 0.020; 3 months: 33.8 (27.2–49.4) versus 54.7 (42.6–64.9) *P* = 0.041; 6 months: 31.1 (24.0–40.6) versus 54.3 (40.8–66.0) *P* = 0.003; 12 months: 29.0 (22.2–37.5) versus 58.0 (39.2–70.5) *P* = 0.004; and 24 months: 26.0 (17.0–35.0) versus 56.5 (44.0–69.5) *P* = 0.002 (Figure 3a). Furthermore, the eGFR slope in this subgroup differed considerably compared to those without retained sC5b-9 generation (−0.31, [IQR −0.56 to −0.20] and 0.19 [IQR −0.13 to 0.39), *P* = 0.007). Two patients had missing data on 30-minute sC5b-9 levels and were excluded from the retained sC5b-9 analyses (both LD-KT). Cases with graft failure were assigned an eGFR value of 10 ml/min per 1.73 m^2^ for the remaining follow-up. Two patients with retained sC5b-9 generation were excluded from the slope analyses due to patient death (DD_CS_-KT, day 64) and loss to follow-up due to emigration (LD-KT, day 69).

To assess the clinical impact of HMP, the eGFR slopes within DD-KT were also compared (Figure 3b). Here, the eGFR slope from 1 to 24 months differed between DD_CS_-KT cases with and without retained sC5b-9 generation (−0.48 [IQR −0.56 to −0.31] vs. 0.13 [IQR −0.09 to 0.51], *P* = 0.014). However, there was no difference in eGFR slopes when comparing DD_HMP_-KT to DD_CS_-KT with retained sC5b-9 generation (0.15 [IQR −0.36 to 0.37] vs. −0.48 [IQR −0.56 to −0.31], *P* = 0.070), as well as between DD_HMP_-KT and DD_CS_-KT without retained sC5b-9 generation (0.15 [IQR −0.36 to 0.37] vs. 0.13 [IQR −0.09 to 0.51], *P* = 1.00). The same was true when excluding the only patient in DD_HMP_-KT with retained sC5b-9 generation.

**Figure 4:**
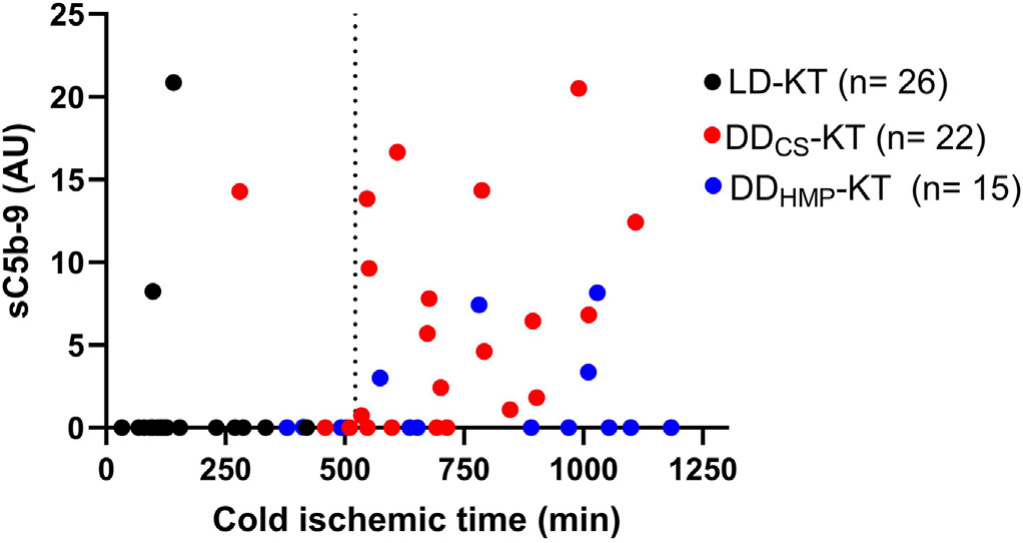
Scatterplot presenting levels of sC5b-9 by cold ischemic time. A dashed line denotes 522.4 minutes on the x-axis. DD_CS_, deceased donor kidney preserved with static cold storage; DD_HMP_, deceased donor kidney preserved with hypothermic machine perfusion; LD, living donor.

To evaluate the association between CIT and IIIS activation in response to IRI, a receiver operating characteristic analysis was performed showing a cutoff threshold for CIT of 522.4 minutes (73% specificity, 83% sensitivity) for prompt generation of sC5b-9 at 1-minute postreperfusion (Figure 4).

## Discussion

In this cohort study of uremic patients undergoing KT, we utilized for the first time an extensive panel of activation markers of IIIS to uncover a simultaneous and prompt activation of the complement, coagulation, and kallikrein-kinin cascade systems following reperfusion, indicating a thrombo-inflammatory response to IRI. This complex inflammatory response was predominantly observed in patients receiving DD_CS_ kidneys, as the local generation of sC5b-9 was nearly absent in the LD-KT population (Figure 1). The simultaneous and consecutive measurements of key activation products of the blood cascade systems enabled us to further investigate the correlations within and between the proteolytic cascades. Apart from the strong interactions within each individual system, we found changes in cross-cascade correlations over time that occurred promptly postreperfusion. For instance, although complement activation product sC5b-9 showed no correlations with other plasma proteins at baseline in DD_CS_ kidneys, it became promptly associated with activation products of both coagulation (FXIa, thrombin) and kallikrein-kinin systems (FXIIa, KK) after reperfusion. These strong cross-cascade correlations endured throughout the sampling period but were completely absent in LD-KT (Figure 2).

Considering these findings, in conjunction with the inherently low exposure to graft ischemia within the LD-KT population, we argued that the prompt thrombo-inflammatory response in DD_CS_ kidneys is a direct consequence of increased graft immunogenicity evoked by early events during DD management, procurement, and organ preservation. Ischemia is incumbent on these early events and a known risk factor for early graft dysfunction and premature graft loss in the DD-KT population.^20^, ^21^, ^22^, ^23^, ^24^, ^25^

To further elucidate the relationship between IRI-induced thromboinflammation and ischemia, the relationship between CIT and sC5b-9 generation was investigated. Soluble C5b-9 is widely recognized as a robust marker of complement activation. However, in the context of IRI, the concurrent generation of sC5b-9 alongside its strong association with other activation markers of IIIS prompted us to recognize sC5b-9 as a response marker of thromboinflammation. We found that a CIT cutoff threshold of 522.4 minutes (73% specificity, 83% sensitivity) was associated with generation of sC5b-9 postreperfusion (Figure 4). Notably, the median CIT in the DD-KT population was restricted to 11 hours and 32 minutes, which is below previously proposed risk threshold values for inferior transplant outcomes.^26^^,^^27^ Admittedly, the overall low exposure to CIT may explain the comparable overall graft-survival and patient survival between the LD-KT and DD-KT populations in our study. Nevertheless, we found that the generation of sC5b-9 was associated with DGF in DD-KT patients, even with a supposedly low burden of ischemia. Furthermore, we noticed that retained generation of sC5b-9 at 30 minutes postreperfusion was associated with mid-term (24 months) allograft dysfunction (Figure 3a). Indeed, patients with retained sC5b-9 generation belonged to the subpopulation experiencing a progressive decline in eGFR, whereas in patients with no retained sC5b-9 generation, mid-term kidney function was preserved. Given these findings, it is conceivable to assume a proportional relationship between the duration of CIT and the risk increment for an injurious thrombo-inflammatory response following reperfusion. The relationship between graft injury and CIT, even in cases of relatively shorter CIT durations, is not a new discovery. A recent meta-analysis focusing on LD-KT revealed that a CIT exceeding 4 hours increased the risk of DGF and has a detrimental effect on both 1-year and 5-year graft survival.^28^

HMP has proven to be superior to static cold storage in preservation of kidneys from both expanded criteria and donation-after-circulatory-death donors.^29^, ^30^, ^31^, ^32^ Preclinical evidence suggests that HMP might reduce the immunogenicity of kidney grafts by clearance of cell debris and toxic metabolites, optimization of renal vascularization, and active preservation of endothelial integrity.^6^^,^^33^, ^34^, ^35^, ^36^ Interestingly, HMP exhibited an attenuation of the thrombo-inflammatory response to IRI, as evidenced by the disappearance of cross-cascade correlations at 10 and 30 minutes, in contrast to cold stored DD kidneys (Figure 2). Moreover, this attenuation was associated with preserved eGFR over a 24-month follow-up period (Figure 3b). Notably, the homogeneity in kidney donor risk index and CIT among DD kidneys undergoing both preservation modalities adds further interest to these findings.

Limited clinical studies have investigated the initial engagement of IIIS in response to IRI, with a specific focus on individual cascade systems. For example, the complement system, assessed by sC5b-9 generation, has been shown to be promptly activated postreperfusion and found to be predictive for DGF and graft dysfunction at 1 year.^11^^,^^37^ Instant kallikrein-kinin system activation during liver transplant surgery has been linked to hemodynamic instability postreperfusion.^38^ In addition, in liver recipients, prompt activation of the coagulation system has been reported postreperfusion.^39^ Our study expands upon current knowledge by providing evidence from human transplant recipients with IRI of an intricate and comprehensive inflammatory response engaging all proteolytic cascades of the IIIS following IRI. This finding carries important clinical implications because it may explain the limited effectiveness observed in clinical trials that exclusively focus on targeting individual components of the IIIS, such as complement therapeutics, to mitigate IRI.^12^, ^13^, ^14^

Interestingly, a clinical trial addressing IRI after DD-KT has shown promise in improving long-term transplant outcomes by using C1INH, a protease-inhibitor that effectively targets key components of the complement, kallikrein-kinin, and coagulation cascades.^40^ Another recent study in a nonhuman primate model adopted a more comprehensive approach, combining C1INH with the anticoagulant heparin, resulting in a substantial reduction in DGF and systemic cytokine release following KT.^41^ Although the authors primarily attribute their success to complement system blockade, their findings challenge this assumption and align with our own, suggesting that therapeutic strategies targeting thromboinflammation may surpass limitations associated with interventions focused on individual cascade systems.

In our study, the lack of pathway-specific biomarkers, apart from MASP1 and MASP2, limits the precise identification of the underlying pathway responsible for IRI-induced thromboinflammation. Additional studies and pathway-specific biomarkers will be essential in uncovering the precise cascade activation sequence. Nevertheless, in DD_CS_ kidneys, the strong correlation between the generation of MASP2 and other markers of IIIS including sC5b-9, FXI, FXII and KK indicated a potential involvement of the lectin pathway. This notion finds support in preclinical evidence showing abnormal L-fucose expression on tubule cells in kidneys exposed to ischemia, inducing autonomous binding to collectin-11, and activating the lectin pathway via MASP-2 interaction following reperfusion.^42^

The study has points for critical discussion. Considering the single-center study design and relatively small patient population, we did not perform multiple testing for all study variables to prevent spurious associations. Instead, the generation of sC5b-9 was analyzed as a response biomarker of IRI-induced thromboinflammation. This consideration was conceivable due to the strong correlations of sC5b-9 to other key components of the proteolytic cascades postreperfusion. Next, the study population did not include donation-after-circulatory-death donors, a modality that was implemented at our center after the study period. Combined with a restricted overall CIT, the IIIS response to more severe ischemic injury could not be investigated. Furthermore, kidney biopsies for parallel histological evaluations of tissue inflammation were not performed.

Nevertheless, we present a high-resolution investigation of the IIIS suggesting that IRI can induce a prompt and interlinked activation of all proteolytic cascade systems involved in the IIIS, leading to a harmful thrombo-inflammatory response. This indicates that individual components, such as the complement system, are part of a larger and more intricate response to IRI rather than being the sole primary effector system. Moreover, the generation of sC5b-9, in addition to serving as a complement activation marker, may also reflect the thrombo-inflammatory response triggered by IRI, because it strongly correlates with activation markers of other cascade systems. Lastly, the efficacy of HMP to mitigate IRI-induced thromboinflammation underscores the importance of preemptive interventions aimed at reducing graft immunogenicity before allograft reperfusion.

## Disclosure

All the authors declare no competing interests. No AI-associated technologies were used in the writing process.

## Supplementary Material

Supplementary File (PDF)

**Table S1.** Multiple comparisons analysis of variance of aligned rank-transformed data for analysis of relationships between IIIS activation markers and the 2 independent variables time and modality. Data are shown as median (1st–3rd quartile).

**Tables S2A–4D.** Assessment of the relationship between activation markers of IIIS within and across the proteolytic cascade systems at all sampling time points for LD-KT (S2a-d), DD_CS_-KT (S3a-d), and DD_HMP_-KT (S4a–d) populations.

**STROBE Statement.**
