# Supplementary material for: Prompt Thrombo-Inflammatory Response to Ischemia-Reperfusion Injury and Kidney Transplant Outcomes
Source: Kidney Int Rep. 2023 Sep 24;8(12):2592–602. doi: 10.1016/j.ekir.2023.09.025 (PMC10719603; doi:10.1016/j.ekir.2023.09.025)
Supplement: Supplementary File (PDF) [file mmc1.pdf]

Modified STROBE Statement—checklist of items that should be included in reports of observational studies (Cohort/Cross-sectional and case-control studies)

	Item No	Recommendation
**Title and abstract**	1	(*a*) Indicate the study's design with a commonly used term in the title or the abstract<br><br>Page 2
		(*b*) Provide in the abstract an informative and balanced summary of what was done and what was found<br><br>Page 2
**Introduction**		
Background/rationale	2	Explain the scientific background and rationale for the investigation being reported<br><br>Pages 3,4
Objectives	3	State specific objectives, including any prespecified hypotheses<br><br>Pages 4
**Methods**		
Study design	4	Present key elements of study design early in the paper<br><br>Pages 4,5
Setting	5	Describe the setting, locations, and relevant dates, including periods of recruitment, exposure, follow-up, and data collection<br><br>Pages 4,5,6
Participants	6	*(a) Cohort study*—Give the eligibility criteria, and the sources and methods of selection of participants. Describe methods of follow-up<br><br>Pages 4,5<br><br>*Case-control study*—Give the eligibility criteria, and the sources and methods of case ascertainment and control selection. Give the rationale for the choice of cases and controls<br><br>*Cross-sectional study*—Give the eligibility criteria, and the sources and methods of selection of participants
Variables	7	Clearly define all outcomes, exposures, predictors, potential confounders, and effect modifiers. Give diagnostic criteria, if applicable<br><br>Pages 5,6
Data sources/ measurement	8*	For each variable of interest, give sources of data and details of methods of assessment (measurement).<br><br>Page 5,6,7
Bias	9	Describe any efforts to address potential sources of bias

		Pages 5,6
Study size	10	Explain how the study size was arrived at (if applicable)<br><br>N/A
Quantitative variables	11	Explain how quantitative variables were handled in the analyses. If applicable, describe which groupings were chosen and why<br><br>Page 7
Statistical methods	12	(*a*) Describe all statistical methods, including those used to control for confounding<br><br>Page 7
		(*b*) Describe any methods used to examine subgroups and interactions<br><br>Page 7
		(*c*) Explain how missing data were addressed<br><br>Page 11
		(*d*) *Cohort study*—If applicable, explain how loss to follow-up was addressed<br><br>Page 11<br><br>*Case-control study*—If applicable, explain how matching of cases and controls was addressed<br><br>*Cross-sectional study*—If applicable, describe analytical methods taking account of sampling strategy
		(*e*) Describe any sensitivity analyses<br><br>N/A

Results

Participants	13*	(a) Report numbers of individuals at each stage of study—eg numbers potentially eligible, examined for eligibility, confirmed eligible, included in the study, completing follow-up, and analyzed<br><br>Pages 4,11
		(c) Use of a flow diagram<br><br>N/A
Descriptive data	14*	(a) Give characteristics of study participants (eg demographic, clinical, social) and information on exposures and potential confounders<br><br>Page 7,8

		(b) Indicate number of participants with missing data for each variable of interest<br><br>Page 11
		(c) *Cohort study*—Summarise follow-up time (eg, average and total amount)<br><br>Page 5
Outcome data	15*	*Cohort study*—Report numbers of outcome events or summary measures over time<br><br>Pages 8,10,11
		Case-control study—Report numbers in each exposure category, or summary measures of exposure
		Cross-sectional study—Report numbers of outcome events or summary measures
Main results	16	(*a*) Give unadjusted estimates and, if applicable, confounder-adjusted estimates and their precision (eg, 95% confidence interval). Make clear which confounders were adjusted for and why they were included<br><br>Page 7-11
Other analyses	17	Report other analyses done—eg analyses of subgroups and interactions, and sensitivity analyses<br><br>Page 7-11

Discussion

Key results	18	Summarise key results with reference to study objectives<br><br>Page 15-16
Limitations	19	Discuss limitations of the study, taking into account sources of potential bias or imprecision. Discuss both direction and magnitude of any potential bias<br><br>Page 15
Interpretation	20	Give a cautious overall interpretation of results considering objectives, limitations, multiplicity of analyses, results from similar studies, and other relevant evidence<br><br>Pages 13-16
Generalisability	21	Discuss the generalisability (external validity) of the study results<br><br>Pages 14-16

*Give information separately for cases and controls in case-control studies and, if applicable, for exposed and unexposed groups in cohort and cross-sectional studies.

Note: An Explanation and Elaboration article discusses each checklist item and gives methodological background and published examples of transparent reporting. The STROBE checklist is best used in conjunction with this article (freely available on the Web sites of PLoS Medicine at http://www.plosmedicine.org/, Annals of Internal Medicine at http://www.annals.org/, and Epidemiology at http://www.epidem.com/). Information on the STROBE Initiative is available at www.strobe-statement.org.

Expression of key markers of the IIIS by modality and sampling time

Table S1

Multiple comparisons ANOVA of aligned rank transformed data for analysis of relationship between IIIS activation markers and the two independent variables time and modality. Data are shown as median (1st-3rd quartile).

	sC5b-9	FXIa-AT (nM)	FXIa-C1INH	Thrombin-AT	Thrombin-C1INH	KK-AT	KK-C1INH	FXIIa-AT	FXIIa-C1INH	MASP1-AT	MASP1-C1INH	MASP2-AT	MASP2-C1INH	C3a
**LD-KT**														
Baseline	0.00 (0.00-0.00)	0.62 (0.48-1.32)	1.90 (1.05-6.00)	0.63 (0.28-0.96)	1.26 (0.07-4.42)	2.42 (1.67-3.80)	2.07 (1.49-9.86)	1.69 (1.10-3.41)	2.19 (1.04-5.73)	0.21 (0.05-0.34)	0.37 (0.00-0.80)	0.26 (0.16-0.46)	0.69 (0.24-1.26)	778 (616-1012)
1 min	0.00 (0.00-0.00)	0.82 (0.51-1.47)	2.08 (1.05-6.40)	0.83 (0.49-1.24)	1.54 (0.18-4.97)	2.76 (1.80-4.42)	3.86 (1.87-9.37)	1.87 (1.15-4.11)	2.21 (1.09-5.34)	0.22 (0.05-0.38)	0.40 (0.00-0.93)	0.28 (0.19-0.48)	0.63 (0.34-1.16)	848 (685-1057)
10 min	0.00 (0.00-0.00)	0.70 (0.51-1.17)	1.78 (1.11-6.00)	0.92 (0.42-1.38)	0.75 (0.01-3.92)	3.02 (1.69-4.49)	2.56 (1.33-8.67)	1.85 (1.19-4.22)	2.29 (0.66-5.93)	0.18 (0.09-0.36)	0.28 (0.04-0.87)	0.26 (0.20-0.45)	0.58 (0.16-1.05)	958 (758-1093)
30 min	0.00 (0.00-0.00)	0.65 (0.51-0.51)	1.93 (1.11-4.31)	0.59 (0.38-1.15)	1.16 (0.16-3.95)	2.28 (1.72-4.59)	2.37 (1.39-7.63)	1.73 (1.06-3.81)	2.23 (1.08-3.96)	0.17 (0.05-0.39)	0.43 (0.00-0.79)	0.22 (0.14-0.50)	0.56 (0.35-1.17)	708 (523-1037)
**DD_{HMP}-KT**														
Baseline	0.00 (0.00-0.00)	0.95 (0.08-1.98)	3.34 (1.37-14.21)	0.35 (0.00-0.66)	1.75 (0.27-5.79)	1.48 (0.42-1.97)	3.16 (0.00-5.01)	0.86 (0.36-1.42)	2.22 (1.29-5.79)	0.16 (0.00-0.39)	0.61 (0.33-3.36)	0.31 (0.11-0.67)	0.94 (0.53-2.75)	916 (509-1205)
1 min	0.00 (0.00-3.02)	1.16 (0.62-2.53)	5.80 (1.61-12.29)	0.62 (0.16-1.05)	5.86 (0.53-11.48)	1.46 (0.75-3.47)	2.93 (0.70-9.35)	0.94 (0.62-2.57)	1.82 (1.26-8.14)	0.19 (0.03-0.64)	0.57 (0.28-1.82)	0.28 (0.14-0.77)	0.85 (0.50-2.34)	983 (562-1133)
10 min	0.00 (0.00-0.00)	0.86 (0.15-2.02)	3.37 (1.41-11.56)	0.41 (0.00-0.87)	1.40 (0.38-5.48)	1.02 (0.47-2.40)	2.59 (0.00-4.81)	0.71 (0.39-2.20)	2.42 (1.25-4.96)	0.14 (0.00-0.45)	0.63 (0.32-2.58)	0.32 (0.13-0.70)	0.94 (0.39-2.73)	1097 (621-1367)
30 min	0.00 (0.00-0.00)	0.97 (0.62-2.06)	3.29 (1.14-10.87)	0.59 (0.00-0.80)	1.70 (0.42-3.54)	1.62 (0.50-2.28)	2.49 (0.00-4.23)	0.87 (0.43-2.03)	2.27 (1.23-3.54)	0.19 (0.07-0.46)	0.85 (0.15-2.05)	0.36 (0.17-0.67)	1.09 (0.51-2.28)	995 (613-1158)
**DD_{CS}-KT**														
Baseline	0.00 (0.00-0.00)	1.25 (0.65-2.6)	2.17 (1.26-7.76)	0.52 (0.00-1.71)	0.70 (0.06-3.15)	1.90 (0.81-5.12)	2.70 (0.85-4.79)	1.24 (0.68-3.17)	2.11 (0.99-6.42)	0.23 (0.04-0.43)	0.64 (0.37-1.27)	0.48 (0.24-0.65)	1.08 (0.56-1.54)	909 (645-1111)
1 min	5.16 (0.00-12.43)	1.79 (0.86-4.55)	2.45 (1.60-7.41)	1.30 (0.60-3.99)	1.04 (0.72-5.99)	2.55 (1.12-6.49)	4.92 (2.27-15.78)	1.90 (0.97-6.31)	2.35 (1.26-7.80)	0.23 (0.11-0.54)	0.78 (0.43-1.12)	0.62 (0.28-0.79)	1.00 (0.66-1.58)	1099 (662-1400)
10 min	0.00 (0.00-3.15)	1.23 (0.76-3.53)	2.32 (1.21-7.05)	0.96 (0.30-2.81)	0.92 (0.41-9.79)	1.98 (1.01-5.41)	4.53 (0.86-6.99)	1.38 (0.87-3.64)	2.47 (1.15-7.32)	0.29 (0.11-0.46)	0.83 (0.32-1.46)	0.51 (0.28-0.70)	1.02 (0.65-1.86)	1166 (750-1504)
30 min	0.00 (0.00-1.7)	1.20 (0.74-2.41)	2.22 (1.38-6.92)	0.72 (0.07-1.87)	0.73 (0.14-6.11)	1.98 (0.89-5.21)	2.95 (0.62-5.18)	1.23 (0.70-2.43)	2.49 (1.05-7.55)	0.25 (0.04-0.46)	0.68 (0.40-1.27)	0.50 (0.21-0.67)	0.94 (0.60-1.75)	1004 (645-1570)
**ART-ANOVA**														
Time P-value	<0.001	<0.001	<0.001	<0.001	<0.001	<0.001	<0.001	<0.001	<0.001	0.02	0.14	0.12	0.68	0.037
Modality P-value	<0.001	0.005	<0.001	0.16	0.56	0.03	<0.001	0.04	<0.001	0.78	0.10	0.14	0.16	0.11
Interaction P-value	<0.001	<0.001	<0.001	0.07	<0.001	<0.001	<0.001	0.24	<0.001	0.08	0.27	0.35	0.53	0.66

Spearman’s correlation matrices of IIIS markers

Table S2a-4d

Assessment of the relationship between activation markers of IIIS within and across the proteolytic cascade systems at all sampling time points for LD-KT (S2a-d), DD$_{CS}$-KT (S3a-d) and DD$_{HMP}$-KT (S4a-d) populations

Tables S2a-d: LD-KT population

Table S2a: Baseline (before reperfusion)

			FXIa		Thrombin		FXIIa		Kallikrein		MASP-1		MASP-2		sC5b-9	C3a
			AT	C1-INH	AT	C1-INH	AT	C1-INH	AT	C1-INH	AT	C1-INH	AT	C1-INH		
**FXIa**	AT	ρ			0.22	-0.02	-0.25	-0.18	-0.04	0.24	**0.62**	0.02	**0.71**	-0.08	N/A	-0.04
		P-value			ns	ns	ns	ns	ns	ns	**<0.001**	ns	**<0.001**	ns	N/A	ns
	C1-INH	ρ			0.34	**0.77**	-0.28	**0.61**	0.16	0.02	-0.06	**0.51**	-0.03	**0.91**	N/A	-0.20
		P-value			ns	**<0.001**	ns	**0.002**	ns	ns	ns	**0.008**	ns	**<0.001**	N/A	ns
**Thrombin**	AT	ρ					**0.61**	0.20	**0.80**	**0.44**	**0.40**	0.20	**0.45**	0.26	N/A	0.03
		P-value					**<0.001**	ns	**<0.001**	**0.028**	**0.042**	ns	**0.02**	ns	N/A	ns
	C1-INH	ρ					**0.43**	**0.87**	0.33	0.13	-0.18	**0.43**	-0.12	**0.74**	N/A	-0.19
		P-value					**0.036**	**<0.001**	ns	ns	ns	**0.035**	ns	**<0.001**	N/A	ns
**FXIIa**	AT	ρ							**0.90**	**0.42**	-0.17	-0.16	-0.02	0.19	N/A	0.02
		P-value							**<0.001**	**0.038**	ns	ns	ns	ns	N/A	ns
	C1-INH	ρ							0.38	0.30	-0.20	**0.52**	-0.22	**0.71**	N/A	-0.11
		P-value							ns	ns	ns	**0.009**	ns	**<0.001**	N/A	ns
**Kallikrein**	AT	ρ									0.07	0.08	0.17	0.10	N/A	-0.02
		P-value									ns	ns	ns	ns	N/A	ns
	C1-INH	ρ									0.23	-0.04	0.28	0.03	N/A	0.15
		P-value									ns	ns	ns	ns	N/A	ns
**MASP-1**	AT	ρ											**0.90**	0.02	N/A	-0.19
		P-value											**<0.001**	ns	N/A	ns
	C1-INH	ρ											0.18	**0.75**	N/A	-0.25
		P-value											ns	**<0.001**	N/A	ns
**MASP-2**	AT	ρ													N/A	-0.24
		P-value													N/A	ns
	C1-INH	ρ													N/A	-0.25
		P-value													N/A	ns
**sC5b-9**		ρ														N/A
		P-value														N/A
**C3a**		ρ														
		P-value														

Table S2b: 1 minute postreperfusion

			FXIa		Thrombin		FXIIa		Kallikrein		MASP-1		MASP-2		sC5b-9	C3a
			AT	C1-INH	AT	C1-INH	AT	C1-INH	AT	C1-INH	AT	C1-INH	AT	C1-INH		
**FXIa**	AT	ρ			0.27	-0.03	-0.22	-0.09	0.04	0.14	**0.65**	0.10	**0.79**	-0.02	N/A	0.02
		P-value			ns	ns	ns	ns	ns	ns	**<0.001**	ns	**<0.001**	ns	N/A	ns
	C1-INH	ρ			**0.47**	**0.78**	0.29	**0.54**	0.20	0.19	-0.04	**0.51**	0.09	**0.89**	N/A	-0.17
		P-value			**0.016**	**<0.001**	ns	**0.007**	ns	ns	ns	**0.007**	ns	**<0.001**	N/A	ns
**Thrombin**	AT	ρ					**0.58**	0.32	**0.78**	**0.44**	**0.39**	0.27	**0.53**	0.36	N/A	-0.20
		P-value					**0.002**	ns	**<0.001**	**0.030**	**0.048**	ns	**0.006**	ns	N/A	ns
	C1-INH	ρ					**0.44**	**0.82**	0.30	0.30	-0.17	**0.45**	-0.07	**0.76**	N/A	-0.21
		P-value					**0.032**	**<0.001**	ns	ns	ns	**0.028**	ns	**<0.001**	N/A	ns
**FXIIa**	AT	ρ							**0.89**	**0.45**	-0.21	-0.16	-0.04	0.20	N/A	-0.31
		P-value							**<0.001**	**0.023**	ns	ns	ns	ns	N/A	ns
	C1-INH	ρ							**0.43**	**0.50**	-0.13	**0.46**	-0.04	**0.65**	N/A	-0.20
		P-value							**0.036**	**0.014**	ns	**0.023**	ns	**0.001**	N/A	ns
**Kallikrein**	AT	ρ									0.11	-0.06	0.27	0.11	N/A	-0.28
		P-value									ns	ns	ns	ns	N/A	ns
	C1-INH	ρ									0.34	0.15	0.37	0.18	N/A	-0.16
		P-value									ns	ns	ns	ns	N/A	ns
**MASP-1**	AT	ρ											**0.92**	0.04	N/A	-0.002
		P-value											**<0.001**	ns	N/A	ns
	C1-INH	ρ											0.32	**0.76**	N/A	-0.07
		P-value											ns	**<0.001**	N/A	ns
**MASP-2**	AT	ρ													N/A	-0.04
		P-value													N/A	ns
	C1-INH	ρ													N/A	-0.22
		P-value													N/A	ns
**sC5b-9**		ρ														N/A
		P-value														N/A
**C3a**		ρ														
		P-value														

Table S2c: 10 minutes postreperfusion

			FXIa		Thrombin		FXIIa		Kallikrein		MASP-1		MASP-2		sC5b-9	C3a
			AT	C1-INH	AT	C1-INH	AT	C1-INH	AT	C1-INH	AT	C1-INH	AT	C1-INH		
**FXIa**	AT	ρ			0.27	0.07	-0.19	-0.11	0.03	0.40	**0.75**	0.004	**0.88**	-0.12	N/A	0.32
		P-value			ns	ns	ns	ns	ns	ns	**<0.001**	ns	**<0.001**	ns	N/A	ns
	C1-INH	ρ			**0.57**	**0.74**	0.33	**0.54**	0.30	0.24	-0.04	**0.56**	0.05	**0.87**	N/A	-0.13
		P-value			**0.003**	**<0.001**	ns	**0.008**	ns	ns	ns	**0.003**	ns	**<0.001**	N/A	ns
**Thrombin**	AT	ρ					**0.68**	0.41	**0.76**	**0.49**	0.29	0.35	**0.43**	**0.45**	N/A	-0.06
		P-value					**<0.001**	ns	**<0.001**	**0.019**	ns	ns	**0.033**	**0.025**	N/A	ns
	C1-INH	ρ					ns	**0.83**	**0.44**	**0.50**	0.01	**0.67**	0.06	**0.72**	N/A	-0.11
		P-value					ns	**<0.001**	**0.036**	**0.019**	ns	**<0.001**	ns	**<0.001**	N/A	ns
**FXIIa**	AT	ρ							**0.92**	**0.44**	-0.23	-0.06	-0.05	0.23	N/A	-0.15
		P-value							**<0.001**	**0.036**	ns	ns	ns	ns	N/A	ns
	C1-INH	ρ							**0.46**	**0.62**	-0.01	**0.69**	-0.08	**0.59**	N/A	-0.16
		P-value							**0.028**	**0.002**	ns	**<0.001**	ns	**0.003**	N/A	ns
**Kallikrein**	AT	ρ									-0.02	-0.01	0.16	0.15	N/A	-0.13
		P-value									ns	ns	ns	ns	N/A	ns
	C1-INH	ρ									-0.33	-0.16	0.37	0.14	N/A	-0.10
		P-value									ns	ns	ns	ns	N/A	ns
**MASP-1**	AT	ρ											**0.87**	0.04	N/A	-0.002
		P-value											**<0.001**	ns	N/A	ns
	C1-INH	ρ											0.15	**0.80**	N/A	-0.06
		P-value											ns	**<0.001**	N/A	ns
**MASP-2**	AT	ρ													N/A	0.14
		P-value													N/A	ns
	C1-INH	ρ													N/A	-0.09
		P-value													N/A	ns
**sC5b-9**		ρ														N/A
		P-value														N/A
**C3a**		ρ														
		P-value														

Table S2d: 30 minutes postreperfusion

			FXIa		Thrombin		FXIIa		Kallikrein		MASP-1		MASP-2		sC5b-9	C3a
			AT	C1-INH	AT	C1-INH	AT	C1-INH	AT	C1-INH	AT	C1-INH	AT	C1-INH		
**FXIa**	AT	ρ			0.36	0.02	-0.14	0.03	0.04	0.12	**0.65**	0.21	**0.822**	0.02	N/A	0.20
		P-value			ns	ns	ns	ns	ns	ns	**<0.001**	ns	**<0.001**	ns	N/A	ns
	C1-INH	ρ			0.38	**0.73**	0.21	**0.50**	0.14	-0.08	-0.11	**0.42**	0.01	**0.88**	N/A	-0.16
		P-value			ns	**<0.001**	ns	**0.019**	ns	ns	ns	**0.039**	ns	**<0.001**	N/A	ns
**Thrombin**	AT	ρ					**0.64**	0.27	**0.77**	0.33	0.39	0.23	**0.62**	0.32	N/A	-0.09
		P-value					**<0.001**	ns	**<0.001**	ns	ns	ns	**0.002**	ns	N/A	ns
	C1-INH	ρ					**0.43**	**0.82**	0.38	0.36	-0.20	0.39	-0.06	**0.68**	N/A	-0.2
		P-value					**0.041**	**<0.001**	ns	ns	ns	ns	ns	**<0.001**	N/A	ns
**FXIIa**	AT	ρ							**0.93**	0.38	-0.19	-0.23	0.02	0.16	N/A	-0.12
		P-value							**<0.001**	ns	ns	ns	ns	ns	N/A	ns
	C1-INH	ρ							**0.43**	**0.44**	-0.15	**0.44**	-0.11	**0.58**	N/A	-0.2
		P-value							**0.046**	**0.045**	ns	**0.042**	ns	**0.005**	N/A	ns
**Kallikrein**	AT	ρ									0.05	-0.13	0.27	0.12	N/A	-0.11
		P-value									ns	ns	ns	ns	N/A	ns
	C1-INH	ρ									0.23	0.02	0.32	-0.01	N/A	-0.02
		P-value									ns	ns	ns	ns	N/A	ns
**MASP-1**	AT	ρ											**0.93**	0.02	N/A	0.04
		P-value											**<0.001**	ns	N/A	ns
	C1-INH	ρ											0.32	**0.68**	N/A	-0.31
		P-value											ns	**<0.001**	N/A	ns
**MASP-2**	AT	ρ													N/A	0.09
		P-value													N/A	ns
	C1-INH	ρ													N/A	-0.22
		P-value													N/A	ns
**sC5b-9**		ρ														N/A
		P-value														N/A
**C3a**		ρ														
		P-value														

S3a-d: DD_{CS}-KT population

Table S3a: Baseline (before reperfusion)

			FXIa		Thrombin		FXIIa		Kallikrein		MASP-1		MASP-2		sC5b-9	C3a
			AT	C1-INH	AT	C1-INH	AT	C1-INH	AT	C1-INH	AT	C1-INH	AT	C1-INH		
**FXIa**	AT	ρ			**0.70**	**0.60**	**0.72**	0.42	**0.66**	0.32	0.42	0.16	**0.70**	0.28	0.07	0.07
		P-value			**<0.001**	**0.004**	**<0.001**	ns	**0.001**	ns	ns	ns	**<0.001**	ns	ns	ns
	C1-INH	ρ			0.13	**0.62**	0.20	**0.59**	0.06	0.05	0.17	**0.61**	0.34	**0.88**	0.10	-0.25
		P-value			ns	**0.003**	ns	**0.006**	ns	ns	ns	**0.002**	ns	**<0.001**	ns	ns
**Thrombin**	AT	ρ					**0.97**	0.26	**0.97**	**0.65**	0.39	-0.09	**0.61**	0.001	0.22	0.27
		P-value					**<0.001**	ns	**<0.001**	**0.002**	ns	ns	**0.002**	ns	ns	ns
	C1-INH	ρ					**0.55**	**0.68**	0.45	**0.50**	-0.01	0.42	0.20	**0.51**	0.28	0.24
		P-value					**0.009**	**0.001**	0.042	**0.026**	ns	ns	ns	**0.020**	ns	ns
**FXIIa**	AT	ρ							**0.98**	**0.59**	0.37	-0.10	**0.63**	0.06	0.17	0.22
		P-value							**<0.001**	**0.007**	ns	ns	**0.002**	ns	ns	ns
	C1-INH	ρ							0.18	0.18	0.22	**0.64**	0.41	**0.52**	0.26	0.19
		P-value							ns	ns	ns	**0.002**	ns	**0.018**	ns	ns
**Kallikrein**	AT	ρ									0.42	-0.20	**0.64**	-0.07	0.17	0.28
		P-value									ns	ns	**0.002**	ns	ns	ns
	C1-INH	ρ									0.37	-0.13	0.31	-0.09	-0.01	**0.45**
		P-value									ns	ns	ns	ns	ns	**0.047**
**MASP-1**	AT	ρ											**0.72**	-0.02	0.07	0.12
		P-value											**<0.001**	ns	ns	ns
	C1-INH	ρ											0.22	**0.76**	0.20	-0.10
		P-value											ns	**<0.001**	ns	ns
**MASP-2**	AT	ρ													-0.04	0.06
		P-value													ns	ns
	C1-INH	ρ													0.10	-0.34
		P-value													ns	ns
**sC5b-9**		ρ														-0.31
		P-value														ns
**C3a**		ρ														
		P-value														

Table S3b: 1 minute postreperfusion

			FXIa		Thrombin		FXIIa		Kallikrein		MASP-1		MASP-2		sC5b-9	C3a
			AT	C1-INH	AT	C1-INH	AT	C1-INH	AT	C1-INH	AT	C1-INH	AT	C1-INH		
**FXIa**	AT	ρ			**0.87**	**0.67**	**0.73**	**0.47**	**0.67**	**0.60**	**0.44**	0.28	**0.83**	0.39	**0.57**	0.23
		P-value			**<0.001**	**0.001**	**<0.001**	**0.038**	**0.001**	**0.005**	**0.040**	ns	**<0.001**	ns	**0.005**	ns
	C1-INH	ρ			0.39	**0.56**	0.13	**0.69**	0.02	0.00	0.13	**0.57**	**0.47**	**0.84**	0.32	-0.15
		P-value			ns	**0.011**	ns	**0.001**	ns	ns	ns	**0.007**	**0.030**	**<0.001**	ns	ns
**Thrombin**	AT	ρ					**0.85**	**0.52**	**0.81**	**0.70**	0.42	0.29	**0.78**	0.32	**0.53**	**0.47**
		P-value					**<0.001**	**0.019**	**<0.001**	**0.001**	ns	ns	**<0.001**	ns	**0.012**	**0.029**
	C1-INH	ρ					**0.61**	**0.87**	**0.45**	**0.54**	0.02	**0.46**	0.44	**0.51**	0.39	0.34
		P-value					**0.004**	**<0.001**	**0.048**	**0.013**	ns	**0.034**	ns	**0.021**	ns	ns
**FXIIa**	AT	ρ							**0.95**	**0.82**	0.29	-0.04	**0.61**	0.06	**0.54**	**0.50**
		P-value							**<0.001**	**<0.001**	ns	ns	**0.002**	ns	**0.010**	**0.019**
	C1-INH	ρ							0.25	0.44	0.18	**0.51**	0.42	**0.45**	0.36	0.19
		P-value							ns	ns	ns	**0.023**	ns	**0.047**	ns	ns
**Kallikrein**	AT	ρ									0.36	-0.11	**0.61**	-0.07	**0.54**	**0.54**
		P-value									ns	ns	**0.003**	ns	**0.009**	**0.010**
	C1-INH	ρ									0.33	0.08	**0.46**	-0.07	0.43	0.33
		P-value									ns	ns	**0.041**	ns	ns	ns
**MASP-1**	AT	ρ											**0.67**	0.17	0.24	0.31
		P-value											**0.001**	ns	ns	ns
	C1-INH	ρ											0.30	**0.79**	0.23	0.03
		P-value											ns	**<0.001**	ns	ns
**MASP-2**	AT	ρ													**0.65**	0.28
		P-value													**0.001**	ns
	C1-INH	ρ													0.28	-0.13
		P-value													ns	ns
**sC5b-9**		ρ														0.29
		P-value														ns
**C3a**		ρ														
		P-value														

Table S3c: 10 minutes postreperfusion

			FXIa		Thrombin		FXIIa		Kallikrein		MASP-1		MASP-2		sC5b-9	C3a
			AT	C1-INH	AT	C1-INH	AT	C1-INH	AT	C1-INH	AT	C1-INH	AT	C1-INH		
**FXIa**	AT	ρ			**0.80**	**0.65**	**0.75**	**0.45**	**0.55**	0.44	**0.48**	0.31	**0.72**	0.40	**0.49**	0.31
		P-value			**<0.001**	**0.002**	**<0.001**	**0.048**	**0.009**	ns	**0.025**	ns	**<0.001**	ns	**0.020**	ns
	C1-INH	ρ			0.26	**0.72**	0.21	**0.75**	-0.06	0.03	0.28	**0.67**	**0.60**	**0.87**	0.30	-0.08
		P-value			ns	**<0.001**	ns	**<0.001**	ns	ns	ns	**0.001**	**0.004**	**<0.001**	ns	ns
**Thrombin**	AT	ρ					**0.89**	0.30	**0.79**	**0.56**	**0.49**	0.07	**0.73**	0.17	**0.44**	**0.51**
		P-value					**<0.001**	ns	**<0.001**	**0.013**	**0.020**	ns	**<0.001**	ns	**0.042**	**0.014**
	C1-INH	ρ					**0.49**	**0.77**	0.23	0.32	0.17	**0.65**	**0.54**	**0.66**	0.36	0.39
		P-value					**0.027**	**<0.001**	ns	ns	ns	**0.002**	**0.013**	**0.001**	ns	ns
**FXIIa**	AT	ρ							**0.88**	**0.72**	**0.48**	0.05	**0.59**	0.19	**0.43**	**0.48**
		P-value							**<0.001**	**0.001**	**0.024**	ns	**0.004**	ns	**0.044**	**0.024**
	C1-INH	ρ							0.00	0.00	0.28	**0.60**	**0.67**	**0.53**	0.11	0.12
		P-value							ns	ns	ns	**0.005**	**0.001**	**0.016**	ns	ns
**Kallikrein**	AT	ρ									**0.51**	-0.17	**0.53**	-0.04	0.32	0.32
		P-value									**0.016**	ns	**0.011**	ns	ns	ns
	C1-INH	ρ									**0.60**	0.07	0.34	-0.04	**0.62**	-0.02
		P-value									**0.007**	ns	ns	ns	**0.005**	ns
**MASP-1**	AT	ρ											**0.60**	0.10	**0.47**	-0.06
		P-value											**0.003**	ns	**0.026**	ns
	C1-INH	ρ											0.31	**0.77**	0.18	0.00
		P-value											ns	**<0.001**	ns	ns
**MASP-2**	AT	ρ													0.41	0.19
		P-value													ns	ns
	C1-INH	ρ													0.24	-0.03
		P-value													ns	ns
**sC5b-9**		ρ														0.06
		P-value														ns
**C3a**		ρ														
		P-value														

Table S3d: 30 minutes postreperfusion

			FXIa		Thrombin		FXIIa		Kallikrein		MASP-1		MASP-2		sC5b-9	C3a
			AT	C1-INH	AT	C1-INH	AT	C1-INH	AT	C1-INH	AT	C1-INH	AT	C1-INH		
**FXIa**	AT	ρ			**0.65**	0.43	**0.55**	0.43	**0.50**	0.32	**0.50**	-0.01	**0.59**	0.24	0.33	0.17
		P-value			**0.001**	ns	**0.008**	ns	**0.019**	ns	**0.018**	ns	**0.004**	ns	ns	ns
	C1-INH	ρ			-0.03	**0.44**	-0.07	**0.50**	-0.17	-0.19	0.17	**0.46**	0.22	**0.85**	-0.21	-0.03
		P-value			ns	**0.045**	ns	**0.020**	ns	ns	ns	**0.031**	ns	**<0.001**	ns	ns
**Thrombin**	AT	ρ					**0.85**	0.24	**0.86**	**0.72**	0.42	-0.21	**0.58**	0.19	**0.55**	**0.46**
		P-value					**<0.001**	ns	**<0.001**	**<0.001**	ns	ns	**0.004**	ns	**0.009**	**0.031**
	C1-INH	ρ					**0.47**	**0.57**	0.40	**0.46**	-0.02	0.26	-0.01	0.24	0.24	**0.70**
		P-value					**0.032**	**0.007**	ns	**0.041**	ns	ns	ns	ns	ns	**<0.001**
**FXIIa**	AT	ρ							**0.98**	**0.81**	0.41	-0.35	**0.46**	-0.22	**0.57**	**0.48**
		P-value							**<0.001**	**<0.001**	ns	ns	**0.033**	ns	**0.006**	**0.023**
	C1-INH	ρ							0.11	0.34	0.20	**0.65**	0.30	0.38	0.27	0.17
		P-value							ns	ns	ns	**0.001**	ns	ns	ns	ns
**Kallikrein**	AT	ρ									**0.44**	-0.34	**0.50**	-0.29	**0.56**	**0.44**
		P-value									**0.041**	ns	**0.019**	ns	**0.007**	**0.041**
	C1-INH	ρ									0.38	-0.06	0.32	-0.28	**0.56**	0.44
		P-value									ns	ns	ns	ns	**0.011**	ns
**MASP-1**	AT	ρ											**0.78**	0.06	0.32	-0.10
		P-value											**<0.001**	ns	ns	ns
	C1-INH	ρ											0.07	**0.63**	-0.04	-0.14
		P-value											ns	**0.002**	ns	ns
**MASP-2**	AT	ρ													0.34	-0.16
		P-value													ns	ns
	C1-INH	ρ													-0.27	-0.28
		P-value													ns	ns
**sC5b-9**		ρ														0.40
		P-value														ns
**C3a**		ρ														
		P-value														

Table S4a-d: DD_{HMP}-KT population

Table S4a: Baseline (before reperfusion)

			FXIa		Thrombin		FXIIa		Kallikrein		MASP-1		MASP-2		sC5b-9	C3a
			AT	C1-INH	AT	C1-INH	AT	C1-INH	AT	C1-INH	AT	C1-INH	AT	C1-INH		
**FXIa**	AT	ρ			0.46	0.14	**0.63**	-0.18	**0.58**	-0.20	**0.90**	0.19	**0.98**	0.18	N/A	**-0.58**
		P-value			ns	ns	**0.012**	ns	**0.024**	ns	**<0.001**	ns	**<0.001**	ns	N/A	**0.023**
	C1-INH	ρ			0.14	**0.85**	0.34	**0.74**	0.16	0.11	0.16	**0.83**	0.20	**0.90**	N/A	-0.41
		P-value			ns	**<0.001**	ns	**0.002**	ns	ns	ns	**<0.001**	ns	**<0.001**	N/A	ns
**Thrombin**	AT	ρ					**0.88**	-0.23	**0.92**	0.36	0.50	-0.02	0.40	-0.01	N/A	0.03
		P-value					**<0.001**	ns	**<0.001**	ns	ns	ns	ns	ns	N/A	ns
	C1-INH	ρ					0.28	**0.66**	0.13	0.38	0.03	**0.68**	0.11	**0.73**	N/A	-0.11
		P-value					ns	**0.007**	ns	ns	ns	**0.004**	ns	**0.002**	N/A	ns
**FXIIa**	AT	ρ							**0.94**	0.20	**0.70**	0.28	**0.63**	0.27	N/A	-0.14
		P-value							**<0.001**	ns	**0.004**	ns	**0.012**	ns	N/A	ns
	C1-INH	ρ							-0.14	0.22	-0.24	**0.66**	-0.19	**0.73**	N/A	0.03
		P-value							ns	ns	ns	**0.007**	ns	**0.002**	N/A	ns
**Kallikrein**	AT	ρ									**0.62**	0.10	**0.56**	0.06	N/A	-0.03
		P-value									**0.013**	ns	**0.032**	ns	N/A	ns
	C1-INH	ρ									-0.19	-0.07	-0.24	-0.11	N/A	0.43
		P-value									ns	ns	ns	ns	N/A	ns
**MASP-1**	AT	ρ											**0.92**	0.09	N/A	**-0.53**
		P-value											**<0.001**	ns	N/A	**0.042**
	C1-INH	ρ											0.19	**0.95**	N/A	-0.45
		P-value											ns	**<0.001**	N/A	ns
**MASP-2**	AT	ρ													N/A	**-0.53**
		P-value													N/A	**0.041**
	C1-INH	ρ													N/A	-0.40
		P-value													N/A	ns
**sC5b-9**		ρ														N/A
		P-value														N/A
**C3a**		ρ														
		P-value														

Table S4b: 1 minute postreperfusion

			FXIa		Thrombin		FXIIa		Kallikrein		MASP-1		MASP-2		sC5b-9	C3a
			AT	C1-INH	AT	C1-INH	AT	C1-INH	AT	C1-INH	AT	C1-INH	AT	C1-INH		
**FXIa**	AT	ρ			**0.57**	0.08	**0.64**	-0.38	**0.54**	-0.22	**0.93**	0.18	**0.90**	0.12	**0.58**	**-0.55**
		P-value			**0.028**	ns	**0.010**	ns	**0.038**	ns	**<0.001**	ns	**<0.001**	ns	**0.024**	**0.035**
	C1-INH	ρ			0.15	**0.90**	0.30	0.39	0.06	-0.16	0.18	**0.63**	0.02	**0.82**	-0.06	0.48
		P-value			ns	**<0.001**	ns	ns	ns	ns	ns	**0.017**	ns	**<0.001**	ns	ns
**Thrombin**	AT	ρ					**0.96**	-0.19	**0.98**	0.22	**0.75**	0.15	**0.63**	0.18	0.28	**-0.60**
		P-value					**<0.001**	ns	**<0.001**	ns	**<0.001**	ns	**0.012**	ns	ns	**0.018**
	C1-INH	ρ					0.36	**0.55**	0.21	0.19	0.14	**0.62**	0.04	**0.85**	0.05	-0.48
		P-value					ns	**0.035**	ns	ns	ns	**0.014**	ns	**<0.001**	ns	ns
**FXIIa**	AT	ρ							**0.95**	0.12	**0.79**	0.27	**0.70**	0.32	0.30	**-0.65**
		P-value							**<0.001**	ns	**<0.001**	ns	**0.004**	ns	ns	**0.009**
	C1-INH	ρ							-0.17	0.23	-0.34	0.33	-0.41	0.45	-0.21	0.18
		P-value							ns	ns	ns	ns	ns	ns	ns	ns
**Kallikrein**	AT	ρ									**0.73**	0.11	**0.66**	0.13	0.28	-0.51
		P-value									**0.002**	ns	**0.008**	ns	ns	ns
	C1-INH	ρ									-0.03	-0.13	-0.10	-0.08	0.10	0.25
		P-value									ns	ns	ns	ns	ns	ns
**MASP-1**	AT	ρ											**0.87**	0.13	**0.58**	**-0.58**
		P-value											**<0.001**	ns	**0.023**	**0.024**
	C1-INH	ρ											0.18	**0.89**	0.03	**-0.52**
		P-value											ns	**<0.001**	ns	**0.048**
**MASP-2**	AT	ρ													0.51	-0.47
		P-value													ns	ns
	C1-INH	ρ													-0.06	**-0.63**
		P-value													ns	**0.013**
**sC5b-9**		ρ														-0.07
		P-value														ns
**C3a**		ρ														
		P-value														

Table S4c: 10 minutes postreperfusion

			**FXIa**		**Thrombin**		**FXIIa**		**Kallikrein**		**MASP-1**		**MASP-2**		**sC5b-9**	**C3a**
			AT	C1-INH	AT	C1-INH	AT	C1-INH	AT	C1-INH	AT	C1-INH	AT	C1-INH		
**FXIa**	AT	ρ			0.50	-0.14	**0.66**	-0.37	**0.55**	-0.24	**0.88**	0.18	**0.95**	0.16	N/A	-0.26
		P-value			ns	ns	**0.008**	ns	**0.032**	ns	**<0.001**	ns	**<0.001**	ns	N/A	ns
	C1-INH	ρ			0.18	**0,81**	0.38	**0.67**	0.20	-0.03	0.19	**0.79**	0.23	**0.85**	N/A	-0.38
		P-value			ns	**<0.001**	ns	**0.007**	ns	ns	ns	**<0.001**	ns	**<0.001**	N/A	ns
**Thrombin**	AT	ρ					**0.91**	-0.24	**0.91**	0.14	**0.56**	0.20	**0.54**	0.19	N/A	-0.16
		P-value					**<0.001**	ns	**<0.001**	ns	**0.029**	ns	**0.039**	ns	N/A	ns
	C1-INH	ρ					0.09	**0.65**	-0.06	0.14	-0.22	**0.61**	-0.10	**0.73**	N/A	-0.43
		P-value					ns	**0.008**	ns	ns	ns	**0.016**	ns	**0.002**	N/A	ns
**FXIIa**	AT	ρ							**0.95**	0.06	**0.73**	0.38	**0.71**	0.34	N/A	-0.33
		P-value							**<0.001**	ns	**0.002**	ns	**0.003**	ns	N/A	ns
	C1-INH	ρ							-0.20	0.07	-0.41	0.49	-0.33	**0.63**	N/A	-0.26
		P-value							ns	ns	ns	ns	ns	**0.012**	N/A	ns
**Kallikrein**	AT	ρ									**0.61**	0.22	**0.59**	0.14	N/A	-0.23
		P-value									**0.015**	ns	**0.020**	ns	N/A	ns
	C1-INH	ρ									-0.24	-0.18	-0.33	-0.28	N/A	-0.07
		P-value									ns	ns	ns	ns	N/A	ns
**MASP-1**	AT	ρ											**0.87**	0.11	N/A	-0.15
		P-value											**<0.001**	ns	N/A	ns
	C1-INH	ρ											0.27	**0.93**	N/A	-0.44
		P-value											ns	**<0.001**	N/A	ns
**MASP-2**	AT	ρ													N/A	-0.30
		P-value													N/A	ns
	C1-INH	ρ													N/A	-0.50
		P-value													N/A	ns
**sC5b-9**		ρ														N/A
		P-value														N/A
**C3a**		ρ														
		P-value														

Table S4d: 30 minutes postreperfusion

			**FXIa**		**Thrombin**		**FXIIa**		**Kallikrein**		**MASP-1**		**MASP-2**		**sC5b-9**	**C3a**
			AT	C1-INH	AT	C1-INH	AT	C1-INH	AT	C1-INH	AT	C1-INH	AT	C1-INH		
**FXIa**	AT	ρ			0.48	-0.05	**0.60**	-0.37	0.50	-0.16	**0.90**	0.21	**0.94**	0.22	0.12	-0.44
		P-value			ns	ns	**0.018**	ns	ns	ns	**<0.001**	ns	**<0.001**	ns	ns	ns
	C1-INH	ρ			0.18	**0.88**	0.35	**0.58**	0.18	-0.15	0.17	**0.80**	0.20	**0.86**	-0.37	**-0.60**
		P-value			ns	**<0.001**	ns	**0.024**	ns	ns	ns	**<0.001**	ns	**<0.001**	ns	**0.017**
**Thrombin**	AT	ρ					**0.95**	-0.19	**0.98**	0.28	**0.56**	0.14	0.49	0.19	0.13	-0.26
		P-value					**<0.001**	ns	**<0.001**	ns	**0.031**	ns	ns	ns	ns	ns
	C1-INH	ρ					0.05	**0.77**	-0.04	0.04	-0.16	**0.68**	-0.09	**0.75**	-0.43	**-0.59**
		P-value					ns	**<0.001**	ns	ns	ns	**0.005**	ns	**<0.001**	ns	**0.022**
**FXIIa**	AT	ρ							**0.95**	0.15	**0.71**	0.31	**0.61**	0.32	0.12	-0.41
		P-value							**<0.001**	ns	**0.003**	ns	**0.016**	ns	ns	ns
	C1-INH	ρ							-0.20	-0.01	**-0.52**	**0.54**	-0.41	**0.60**	-0.31	-0.19
		P-value							ns	ns	**0.046**	**0.037**	ns	**0.017**	ns	ns
**Kallikrein**	AT	ρ									**0.61**	0.17	**0.52**	0.18	0.19	-0.32
		P-value									**0.017**	ns	**0.046**	ns	ns	ns
	C1-INH	ρ									-0.17	-0.14	-0.24	-0.28	0.00	0.13
		P-value									ns	ns	ns	ns	ns	ns
**MASP-1**	AT	ρ											**0.92**	0.16	0.12	**-0.52**
		P-value											**<0.001**	ns	ns	**0.049**
	C1-INH	ρ											0.23	**0.93**	-0.25	**-0.51**
		P-value											ns	**<0.001**	ns	**0.050**
**MASP-2**	AT	ρ													0.12	**-0.53**
		P-value													ns	**0.045**
	C1-INH	ρ													-0.37	**-0.64**
		P-value													ns	**0.010**
**sC5b-9**		ρ														0.43
		P-value														ns
**C3a**		ρ														
		P-value														
